# Demographic Representation in Clinical Trials for Transthyretin-Associated Cardiac Amyloidosis: A Systematic Review

**DOI:** 10.31083/RCM53698

**Published:** 2026-09-08

**Authors:** Dimitri Ford, Chima Amadi, Akshay Chandora, Matthew E. Gold, Shanelle Brodeur, Ridwan Azees, Nicolas Bakinde, Anekwe E. Onwuanyi, Marshaleen Henriques King

**Affiliations:** ^1^Department of Internal Medicine, Morehouse School of Medicine, Atlanta, GA 30310, USA; ^2^Division of Cardiology, Department of Medicine, Emory School of Medicine, Atlanta, GA 30322, USA; ^3^Division of Cardiology, Department of Medicine, Morehouse School of Medicine, Atlanta, GA 30310, USA; ^4^Division of Pulmonary Critical Care, Department of Medicine, Morehouse School of Medicine, Atlanta, GA 30310, USA

**Keywords:** amyloidosis, cardiomyopathies, clinical trials as topic, health equity, African Americans, health disparities

## Abstract

**Background:**

Underrepresentation of minority groups in clinical trials exacerbates health disparities and limits the generalizability of findings. Transthyretin amyloid cardiomyopathy (ATTR-CM) is a condition that disproportionately affects certain racial and ethnic groups, yet the extent to which trial enrollment reflects disease burden remains unclear. Misalignment between disease prevalence and trial representation may delay treatment development and increase the economic burden associated with late diagnosis and suboptimal management. To evaluate racial and ethnic representation in completed U.S.-based ATTR-CM clinical trials by comparing observed participant enrollment with expected enrollment based on disease prevalence, and to assess whether demographic reporting and representation improved following the 2017 Food and Drug Administration (FDA) Final Rule.

**Methods:**

This systematic review assessed US-based ATTR clinical trials registered on ClinicalTrials.gov through 2025 (search date: February 12, 2025). Only completed trials with publicly available results were included. Demographic data were extracted at the trial level. For each group, an enrollment fraction (EF) was calculated as observed enrollment ÷ expected enrollment, based on the Cardiac Amyloidosis Registry Study (CARS) prevalence; adequacy was defined as EF ≥0.75.

**Results:**

Of the 264 clinical trials on ATTR identified, 16 met the inclusion criteria. African American individuals/individuals of African descent had EFs below the adequacy threshold of 0.75 across all reviewed trial phases compared with their Asian or White counterparts. Following implementation of the FDA Final Rule in 2017, reporting of demographic data increased in the overall study population (from 60% to 83.3%). Although the racial distribution remained significantly different from the expected population distribution in both periods, the enrollment fraction for Black participants declined from 0.291 before 2017 to 0.125 after 2017, with similar decreases observed among other minority groups.

**Conclusions:**

Black individuals remain substantially underrepresented in US-based ATTR-CM clinical trials despite improved demographic reporting after the 2017 FDA Final Rule. Actionable strategies, including community engagement, diversification of trial sites, enrollment targets, and sponsor accountability, are urgently needed to improve representativeness and expand access.

## 1. Introduction

Transthyretin-associated cardiac amyloidosis (ATTR-CM) is a progressive and life-threatening condition. It is caused by the misfolding of transthyretin (TTR) proteins, which aggregate as amyloid fibrils in cardiac tissue and infiltrate the myocardium, leading to restrictive cardiomyopathy, heart failure, and arrhythmias [1]. ATTR-CM exists in two main forms: hereditary transthyretin amyloidosis (ATTRv) and wild-type transthyretin amyloidosis (ATTRwt), the latter typically affecting older adults without known genetic mutations [2]. Median survival following diagnosis ranges from 2 to 6 years, with worse outcomes in those presenting with advanced heart failure. ATTRwt may account for up to 10–15% of heart failure cases with preserved ejection fraction (HFpEF) in older adults, and autopsy studies suggest a prevalence as high as 25% in men over 80 years [3]. Yet, the disease remains underdiagnosed, often mistaken for other forms of cardiomyopathy. This contributes to an average diagnostic delay of 34 months, with worse cardiac function associated with longer delays in diagnosis [4].

Clinical trials play a transformative role in reshaping the care of patients with ATTR-CM. Landmark studies, such as the ATTR-ACT trial, demonstrated that therapies like tafamidis significantly reduce mortality and cardiovascular-related hospitalizations [5]. These advances underscore the critical importance of clinical research in altering the natural history of this once-fatal disease. However, significant challenges persist. Despite advancements in diagnostics and therapeutics, disparities in outcomes remain. Evidence indicates that certain populations, particularly racial and ethnic minorities and lower socioeconomic groups, experience delayed diagnosis, reduced access to advanced therapies, and worse prognosis [6,7]. Although access to clinical trials should be equitable, multiple studies have shown underrepresentation of minority groups. In response to concerns about transparency and equity in research, the US government implemented the Final Rule for Clinical Trials Registration and Results Information Submission (CTRRIS) in 2017. This policy mandates the timely registration and reporting of clinical trial data on ClinicalTrials.gov [8,9]. While this was a step forward in promoting accountability and inclusivity, we hypothesized that a discrepancy still exists between the demographics of patients enrolled in ATTR-CM trials and those most burdened by the disease. Addressing this gap is crucial for ensuring equitable benefit from scientific advances.

## 2. Methods

This was a systematic review using a search on ClinicalTrials.gov on 12 February 2025. The terms “cardiac amyloidosis” and “amyloidosis cardiac” were used to identify the initial studies, and were then filtered to completed, US-based adult studies. No time constraints were utilized. We extracted trial-level data (number enrolled, phase, year, and reported race/ethnicity and sex distributions). Trials were included if they enrolled ATTR-CM patients predominantly and publicly reported demographic results. Trials with pediatric populations or lacking demographic data were excluded.

To quantify representation, the enrollment fraction (EF) was calculated for White and Black participants only, as incidence data for other demographic groups were unavailable. EF, originally described by Murthy et al. [10], is defined as the number of clinical trial enrollees divided by the estimated number of US cases within each race, ethnicity, and sex subgroup.

The formula for calculating EF is as follows:

EF = Observed enrollment / Expected Enrollment

Expected Enrollment = total number of participants enrolled × CARS observed demographic percentage.

Estimates of ATTR prevalence were obtained from the Cardiac Amyloidosis Registry Study (CARS), a multicenter registry established in 2019 that includes patients with transthyretin-related (both wild-type and variant) and light chain (AL) cardiac amyloidosis evaluated at major amyloidosis centers between 1997 and 2023 [11]. Per the CARS observational study, the prevalence of ATTR is as follows: Whites 73%, Black/African American 24%, Asian 2%, other 1%. Prevalence per sex was as follows: Men 87% and women 13%.

An EF threshold of 0.75 was selected to define adequate representation because it reflects near-proportional enrollment while allowing for expected variability in recruitment, eligibility criteria, and registry-based prevalence estimates. This cutoff has been used in prior literature evaluating demographic proportionality in US clinical trials to indicate near-parity with disease prevalence while allowing for expected sampling variability and logistical constraints in recruitment. An EF of 1.0 reflects perfect proportionality between trial enrollment and disease prevalence within a demographic subgroup. However, given inherent fluctuations in registry-based prevalence estimates, trial eligibility criteria, and geographic concentration of specialized centers, a tolerance range of approximately 25% below parity (i.e., EF ≥0.75) is considered methodologically reasonable to represent adequate inclusion. Values below this threshold indicate substantial underrepresentation relative to the disease population, while values exceeding 1.25 denote relative overrepresentation. This convention provides a pragmatic benchmark for assessing equity in trial enrollment and has been adopted in recent diversity analyses of cardiovascular and oncologic clinical research [10,12]. The primary focus of this study was to evaluate demographic representation and reporting characteristics rather than treatment efficacy or clinical outcomes, a formal risk-of-bias assessment was not performed.

Descriptive statistics were generated using Python (3.13.5, Python Software Foundation, Wilmington, DE, USA), and chi-square tests were performed to assess differences in EF across groups. A *p*-value < 0.05 was considered statistically significant.

## 3. Results

The study selection process is shown in Fig. 1. A total of 264 clinical trials on amyloidosis were identified, of which 16 met the inclusion criteria specifically for ATTR. As shown in Table 1, these trials enrolled a total of 11,371 participants, with a male-to-female ratio of approximately 3:1. Among the 16 trials, five were phase 3, four were observational, four were phase 2, one was phase 4, one was a combined phase 1 and 2, and one was an interventional study without a reported phase. Of these, 10 trials were conducted before the implementation of CTRRIS in 2017, and 6 clinical trials were conducted afterward. Prior to 2017, 60% of trials reported racial demographics, while 83.3% reported racial demographics afterwards.

**Fig. 1.**
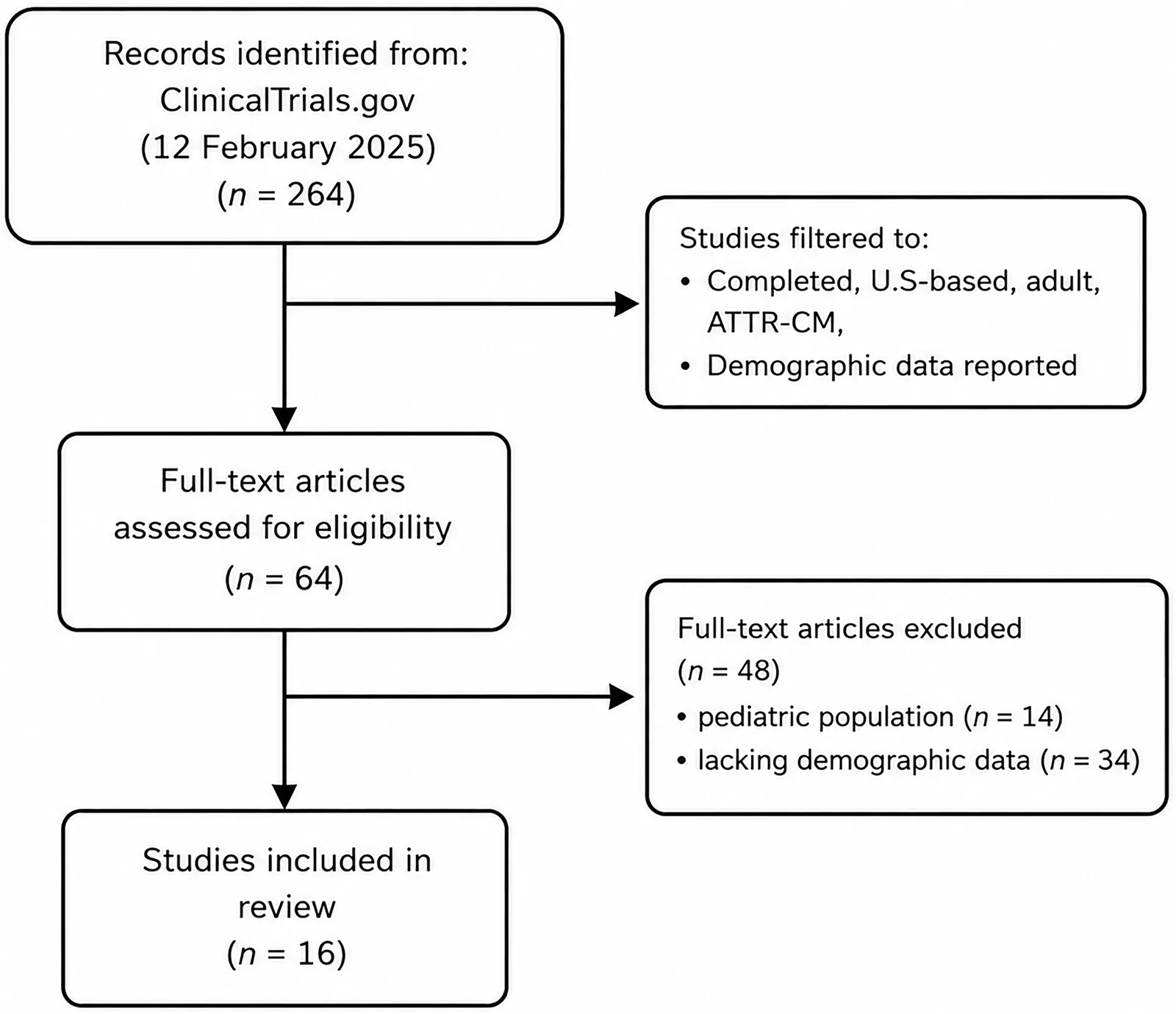
**PRISMA flow diagram of the study selection process**. A total of 264 records were identified through the ClinicalTrials.gov search. Following application of the predefined eligibility criteria and title/abstract screening, 200 records were excluded, leaving 64 full-text articles for eligibility assessment. Of these, 48 were excluded (14 pediatric studies and 34 lacking demographic data), resulting in 16 studies included in the review. PRISMA, Preferred Reporting Items for Systematic Reviews and Meta-Analyses.

**Table 1.** **Comparing participant demographics**.

Name	Year	Study design	Enrollment	Male	Female	Black	White	Asian
Transthyretin Amyloidosis Outcomes Survey (THAOS)	2008	Observational	6718	4304	2414	318	3441	295
The Effects of Fx-1006A on Transthyretin Stabilization and Clinical Outcome Measures in Patients With Non-V30M Transthyretin Amyloidosis	2008	Phase 2	21	13	8	Did not report	Did not report	Did not report
The Effects Of Fx-1006A On Transthyretin Stabilization And Clinical Outcome Measures In Patients With *V122I* Or Wild-Type TTR Amyloid Cardiomyopathy	2008	Phase 2	35	32	3	Did not report	Did not report	Did not report
Safety And Efficacy Evaluation Of Fx-1006a In Patients With *V122i* Or Wild-Type Transthyretin (TTR) Amyloid Cardiomyopathy	2009	Phase 3	31	28	3	4	27	0
Burden of Disease Study In Patients With Transthyretin Familial Amyloidosis Polyneuropathy (TTR-FAP) of Transthyretin Cardiomyopathy (TTR-CM) And Caregivers	2012	Observational	92	57	35	Did not report	Did not report	Did not report
Doxycycline and TUDCA in Patients With Transthyretin Amyloid Cardiomyopathy	2013	Phase 1 and 2	30	29	1	Did not report	Did not report	Did not report
Safety and Efficacy of Tafamidis in Patients With Transthyretin Cardiomyopathy (ATTR-ACT)	2013	Phase 3	441	398	43	63	357	18
A Extension Study to Evaluate Revusiran (ALN-TTRSC) in Patients With Transthyretin (TTR) Cardiac Amyloidosis	2014	Phase 2	25	23	2	4	21	0
ENDEAVOUR: Phase 3 Multicenter Study of Revusiran (ALN-TTRSC) in Patients With Transthyretin (TTR) Mediated Familial Amyloidotic Cardiomyopathy (FAC)	2014	Phase 3	206	158	48	104	95	0
Long-term Safety of Tafamidis in Subjects With Transthyretin Cardiomyopathy	2016	Phase 3	1728	1542	186	159	1468	80
Study of AG10 in Amyloid Cardiomyopathy	2018	Phase 2	49	45	4	10	35	1
Prevalence and Characteristics of Transthyretin Amyloidosis in Patients With Left Ventricular Hypertrophy of Unknown Etiology (TTRACK)	2018	Observational	766	533	233	21	712	27
Efficacy and Safety of AG10 in Subjects With Transthyretin Amyloid Cardiomyopathy	2019	Phase 3	632	570	62	30	555	13
Long-term Monitoring of Patients With Cardiac Amyloidosis With Implantable Event Monitors	2020	Not reported	24	24	0	0	24	0
SGLT2 Inhibitors in Transthyretin Amyloid Cardiomyopathy	2022	Phase 4	15	13	2	2	13	0
A Retrospective Validation Study To Identify Chart-Based Clinical Diagnosis Of Wild-Type Transthyretin Amyloid Cardiomyopathy (ATTRwt-CM) And Non-Amyloid Heart Failure Among Patients With Heart Failure (HF)	2023	Observational	558	558	0	Did not report	Did not report	Did not report
Total			11,371	8327	3044	715	6748	434

Abbreviations: THAOS, Transthyretin Amyloidosis Outcomes Survey; TTR, transthyretin; TTR-FAP, transthyretin familial amyloid polyneuropathy; TTR-CM, transthyretin cardiomyopathy; TUDCA, tauroursodeoxycholic acid; HF, heart failure; ATTRwt-CM, wild-type transthyretin cardiomyopathy; SGLT2, sodium–glucose cotransporter 2.

The overall racial demographic of enrolled participants was as follows: Whites 59.4%, Black or African Americans at 6.3%, Asians (3.8%), Latino individuals (1.9%), Native Hawaiian or Other Pacific Islanders (<0.1%), and Others (1.2%), as shown in Fig. 1. Approximately 27.4% of participants were enrolled in studies that did not report race and/or ethnicity information. The study selection process for the included trials is summarized in the PRISMA flow diagram (Fig. 2).

**Fig. 2.**
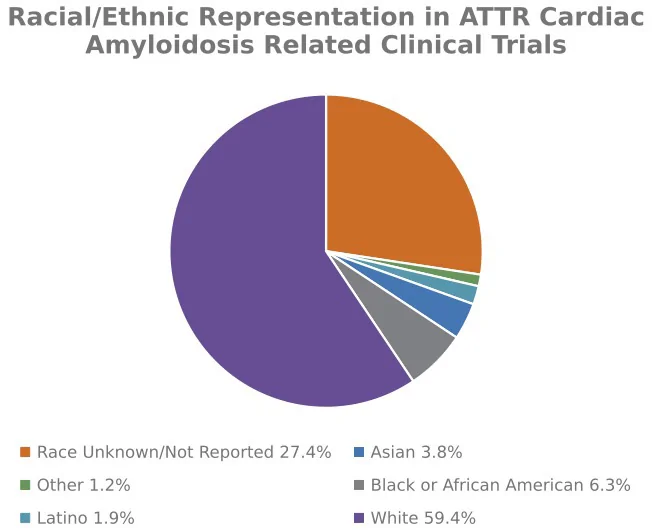
**Race and ethnicity representation in transthyretin associated cardiac Aamyloidosis trials: This chart displays the breakdown of the race unknown/not reported (27.4%) and ethnicity of patients in clinical trials**. Abbreviations: ATTR-CM, transthyretin amyloid cardiomyopathy.

Table 2 shows the EF for each race to be White 0.81 (95% confidence interval (CI) 0.80–0.82), Black/African American 0.26 (95% CI 0.24–0.28), Asian 1.9 (95% CI 1.73–2.08), (*p* value < 0.001) and Men 0.84 (95% CI 0.83–0.85) and Women 2.06 (95% CI 1.99–2.12), (*p* value < 0.001).

**Table 2.** **Overall cardiac amyloidosis enrollment fraction ratio comparison between races**.

Group	Observed	Expected	EF	95% CI
White	6748	8301	0.813	0.798–0.822
Black	715	2729	0.262	0.242–0.278
Asian	434	227	1.912	1.725–2.075
chi-square goodness-of-fit tests: *p* ≤ 0.001				
Men	8327	9893	0.842	0.831–0.849
Women	3044	1478	2.060	1.985–2.115

Abbreviations: EF, enrollment fraction; CI, confidence interval.

Comparison of enrollment fractions before and after implementation of the 2017 FDA Final Rule is shown in Tables 3,4. Prior to 2017, the EF was as follows: White 0.79 (95% CI 0.77–0.81), African American 0.29 (95% CI 0.27–0.31), Asian 2.1 (95% CI 1.87–2.33), (*p* value < 0.001) and Men 0.81 (95% CI 0.79–0.82) and Women 2.25 (95% CI 2.17–2.34), (*p* value < 0.001). After 2017 the EF was as follows: White 0.87 (95% CI 0.84–0.90), African American 0.13 (95% CI 0.11–0.15), Asian 0.98 (95% CI 0.88–1.08), (*p* value < 0.001) and Men 0.96 (95% CI 0.94–0.98) and Women 1.11 (95% CI 1.06–1.16), (*p* value 0.013).

**Table 3.** **Cardiac amyloidosis enrollment fraction ratio comparison between races prior to 2017**.

Group	Observed	Expected	EF	95% CI
White	5409	6820	0.793	0.774–0.806
Black	652	2242	0.290	0.266–0.314
Asian	393	187	2.102	1.868–2.332
chi-square goodness-of-fit tests: *p* ≤ 0.001				
Men	6584	8128	0.810	0.798–0.822
Women	2743	1214	2.259	2.165–2.335

Abbreviations: EF, enrollment fraction; CI, confidence interval.

**Table 4.** **Cardiac amyloidosis enrollment fraction ratio after 2017**.

Group	Observed	Expected	EF	95% CI
White	1339	1526	0.878	0.842–0.898
Black	63	502	0.125	0.108–0.152
Asian	41	42	0.976	0.878–1.082
chi-square goodness-of-fit tests: *p* < 0.001				
Men	1743	1818	0.958	0.939–0.981
Women	301	272	1.107	1.058–1.162
chi-square goodness-of-fit tests: *p* = 0.013				

Abbreviations: EF, enrollment fraction; CI, confidence interval.

For phase 3 and 4 clinical trial EF were found to be White 1.127 (95% CI 1.11–1.13), African American 0.49 (95% CI 0.45–0.53), Asian 1.82 (95% CI 1.56–2.08), (*p* value < 0.001), Men 1.018 (95% CI 0.99–1.01) and Women 0.86 (95% CI 0.79–0.93), *p* value of 0.004 (Table 5).

**Table 5.** **Phase 3 and 4: cardiac amyloidosis enrollment fraction ratio**.

Group	Observed	Expected	EF	95% CI
White	2515	2232	1.127	1.106–1.134
Black	362	734	0.493	0.453–0.527
Asian	111	61	1.820	1.561–2.079
chi-square goodness-of-fit tests: *p* < 0.001				
Men	2709	2660	1.018	0.980–1.058
Women	344	398	0.864	0.790–0.930
chi-square goodness-of-fit tests: *p* = 0.004				

Abbreviations: EF, enrollment fraction; CI, confidence interval.

For other phases (1, 2), the EF was found to be White 0.69 (95% CI 0.67–0.71), African American 0.18 (95% CI 0.15–0.21), Asian 1.93 (95% CI 1.61–2.25), (*p* value < 0.001) and Men 0.77 (95% CI 0.75–0.79) and Women 2.48 (95% CI 2.36–2.60), *p* value < 0.001 (Table 6).

**Table 6.** **Phases 1, and 2: cardiac amyloidosis enrollment fraction ratio**.

Group	Observed	Expected	EF	95% CI
White	4233	6113	0.692	0.668–0.712
Black	353	2010	0.176	0.152–0.208
Asian	323	167	1.934	1.614–2.246
chi-square goodness-of-fit tests: *p* ≤ 0.001				
Men	5618	7285	0.771	0.753–0.787
Women	2700	1089	2.479	2.357–2.603
chi-square goodness-of-fit tests: *p* ≤ 0.001				

Abbreviations: EF, enrollment fraction; CI, confidence interval.

## 4. Discussion

Our systematic review of clinical trials in ATTR-CM demonstrates persistent and substantial differences in racial and ethnic representation. Asian participants were overrepresented (EF of 1.91), whereas White participants demonstrated adequate representation (EF = 0.81), exceeding the predefined adequacy threshold of 0.75 but remaining below the ideal EF of 1.0. By contrast, Black or African American participants exhibited an EF of just 0.26, which represents a 3.11-fold and 7.35-fold lower inclusion than White and Asian participants, respectively. Among sex categories, women had a higher-than-expected (EF = 2.05) relative to registry-derived prevalence estimates, whereas men demonstrated adequate representation (EF = 0.84). Although representation modestly improved in phase 3 trials (31.25% of studies), where Black participants achieved an EF of 0.493, this still fell short of adequacy and highlighted even greater disparities in earlier-phase trials (EF = 0.176).

Despite significant therapeutic breakthroughs and expanded awareness of ATTR-CM, Black individuals, who account for nearly one quarter of the US ATTR-CM population, remain markedly underrepresented in research participation. In contrast, White and Asian participants are consistently overrepresented, while women are modestly overrepresented relative to disease prevalence. Although the 2017 FDA Final Rule improved demographic reporting transparency, it did not translate into improved minority participation. These findings highlight a widening gap between disease burden and research representation, threatening both the external validity of clinical trial findings and the equitable distribution of therapeutic advances.

Over the past decade, therapies such as tafamidis and acoramidis have transformed ATTR-CM, a uniformly fatal condition, to one in which disease modification and prolonged survival are achievable. In addition, diflunisal, an oral nonsteroidal anti-inflammatory drug that stabilizes transthyretin tetramers, has been used off-label as a lower-cost therapeutic option for selected patients. However, it is generally considered most suitable for patients with early-stage disease and preserved renal function as it carries the risk for gastrointestinal bleeds, renal impairment, and fluid retention. However, the clinical benefit of these therapeutic breakthroughs cannot be generalized to populations due to insufficient representation in the pivotal studies that established their efficacy. For example, in the pivotal ATTR-ACT and ATTRibute-CM trials evaluating tafamidis and acoramidis, Black participants accounted for only 14.4% and 4.8% of enrollees, respectively [5,13,14]. These figures fall far short of their estimated disease prevalence of approximately 24% found in the CARS registry [11]. Such imbalances risk limiting therapeutic applicability and may perpetuate disparities in outcomes for groups already bearing disproportionate disease burden.

It is also worth discussing that ATTR-CM has changed considerably over time. Better noninvasive diagnostic tools—including technetium-based cardiac scintigraphy, cardiac MRI, and broader genetic testing—combined with growing clinical awareness, have made it much easier to identify cases that would previously have gone undetected. As a result, current prevalence estimates likely appear higher than those from older studies not because the disease is genuinely more common, but because we are simply better at finding it. When comparing prevalence figures or enrollment fractions from different time periods, diagnostic evolution should be kept in mind.

The persistent underrepresentation of Black patients in ATTR-CM clinical trials reflects a complex interplay of inherited predisposition, social determinants of health, and structural inequities. Genetic epidemiology demonstrates a high prevalence of the pathogenic *V142I* (formerly *V122I*) transthyretin variant among individuals of West African, Afro-Caribbean, and Caribbean-Hispanic ancestry. This variant has been associated with higher risks of developing heart failure among individuals of African and Hispanic ancestry [15]. It’s been further shown that Black patients, particularly those carrying the V142I mutation, face significantly worse event-free survival and elevated incidences of left ventricular assist device implantation, cardiac transplantation, heart failure hospitalization, and all-cause mortality, even after adjustment for disease stage and socioeconomic status (SES) [16]. Socioeconomic disadvantage further compounds this vulnerability. Black patients are disproportionately concentrated in the lowest SES quintiles, and those in high-deprivation neighborhoods experience higher rates of heart failure hospitalization and mortality compared to White patients in similarly disadvantaged contexts [17]. These patterns illustrate that genetic risk is amplified by social inequities, reinforcing the need for early identification strategies among at-risk groups. Large organizations like the American Heart Association have recommended early identification of amyloidosis within at-risk populations such as individuals with Heart failure with preserved ejection fraction (of which the majority comprise Hispanic and Black individuals), individuals of West African origin, or those with small fiber polyneuropathy [18].

Even after effective therapies have been developed, access inequities persist. For instance, tafamidis, the first FDA-approved treatment for ATTR-CM carries an annual list price of approximately $ 225,000 [19]. This cost is nearly four times the median annual household income for Black families ( $ 59,363), compared to White ( $ 93,948) and Asian ( $ 112,800) households. Such cost differentials risk exacerbating existing health inequities, despite proven clinical benefit.

Although the 2017 Final Rule increased demographic reporting from 60% to 83.3%, minority participation paradoxically declined (EF for Black participants decreased from 0.29 to 0.12 post-regulation). In many ways, it failed to confront the deeper structural drivers of racial and socioeconomic underrepresentation in research participation [20]. Meanwhile, White and Asian participants consistently met or exceeded adequacy thresholds of EF >0.75. This disconnect underscores that regulatory transparency alone cannot overcome entrenched barriers rooted in medical mistrust, socioeconomic constraints, and inequitable research infrastructure. Historical ethical violations, such as the Tuskegee Syphilis Study, continue to erode trust in medical research among minority communities. Concurrently, logistical barriers like transportation, financial strain, and limited specialty access, further restrict trial accessibility and participation. Language and cultural discordance between research staff and underrepresented populations further inhibit effective recruitment, particularly when site selection favors predominantly White, affluent regions through convenience sampling. Conversely, the relatively high EFs among Asian participants and women may, in part, reflect sampling biases or registry-based misclassification or a combination of the two causing misrepresentations leading to incorrectly elevated EF values. Asian and women could also be poorly represented within the geographic areas that the tertiary centers contributing to the CARS registry are located.

Addressing these inequities requires a paradigm shift from passive regulatory compliance to proactive, community-engaged strategies. Regulatory reform must be paired with strategies that cultivate trust and accessibility: community partnerships, engaging with local organizations and minority-serving institutions, culturally tailored education, and practical support such as transportation assistance and flexible scheduling. Research teams should reflect the linguistic and cultural diversity of target populations, all of which are critical for cultivating trust and enhancing trial accessibility among historically marginalized populations [21]. Funding agencies should tie trial approval or continuation to measurable diversity benchmarks. The stakes are high: in an era increasingly governed by value-based care, the continued exclusion of racially and socioeconomically diverse populations contributes to diagnostic delays, preventable hospitalizations, and suboptimal application of therapies, thereby worsening health disparities and inflating long-term healthcare costs. Given that Black participants comprise a significant proportion of the US ATTR-CM population and are more likely to present with advanced disease, ensuring their equitable inclusion in clinical trials is not only a moral and ethical obligation, but a strategic necessity for improving population health outcomes and advancing health system efficiency.

In sum, the current landscape of ATTR-CM research reflects deep-rooted inequities in racial and socioeconomic inclusion. Achieving representative participation demands systemic reform, institutional accountability, and sustained community engagement. By embedding expanded access into the structure of clinical research, the scientific community can ensure that emerging therapies are reflective of and available to the populations they are designed to serve. Achieving representative inclusion is essential not only for scientific validity and therapeutic efficacy, but also for advancing health equity in the management of cardiac amyloidosis.

## 5. Limitations

Several limitations should be acknowledged. Approximately 27.4% of the trials did not report racial or ethnic demographic information potentially biasing the interpretation of enrollment. First, ATTR-CM subtype and diagnostic approach were inconsistently documented across studies, which precluded meaningful analysis of whether demographic representation varied by disease subtype or how patients were diagnosed. In addition, our reference point for the observed prevalence of ATTR-CM was obtained using data from a single registry from 1997 to 2023; however, this is only a limited time frame to draw conclusions for proper epidemiological analysis. This database is comprised of data from only 20 tertiary, mostly academic centers, which can exclude a percentage of patients with cardiac amyloidosis, especially from community-based practices. This can affect the generalizability of our analysis given we were unable to compare our trials to true population level prevalence of the disease within various demographics, like in the case of women and Asians. Furthermore, because this analysis relied on publicly available trial-level data rather than individual participant data, complete assessment of potential participant overlap across studies was not possible.

An important limitation of this study is the use of the CARS as the reference population for enrollment fraction calculations. CARS is an exclusively US-based registry, whereas many of the included ATTR clinical trials were multinational and enrolled participants from Europe, Asia, and other regions where the demographic distribution of ATTR-CM may differ substantially from that of the United States. Consequently, the use of CARS as the reference population may influence the calculated enrollment fractions, potentially overestimating representation in some groups while underestimating representation in others. Although analyses restricted to US-based trial populations would provide a more direct comparison, country-specific demographic reporting was infrequently available in published trial data. Therefore, the findings should be interpreted within the context of this limitation.

Additionally, our study relied solely on publicly available data extracted from ClinicalTrials.gov. Race is typically self-reported in trials, which may introduce some misclassification or lack of classification. This is especially true with the Latino population, which is a meaningful subgroup with a known ATTR genetic predisposition, accounted for 1.9% of reported patients in our study but given the lack of CARS data on this population we were unable to calculate an EF for this group. Future studies should integrate multi-database approaches and require standardized, transparent demographic reporting to enable comprehensive equity assessment.

## 6. Conclusion

This study highlights profound and persistent imbalances in racial and ethnic representation within US cardiac amyloidosis clinical trials, particularly the under-enrollment of Black participants. Although the 2017 FDA Final Rule improved demographic reporting, it did not improve enrollment of minorities. Although later phase trials demonstrated modest progress, no phase achieved adequate representation. Ensuring equitable inclusion requires a comprehensive strategy that combines community engagement with targeted recruitment strategies and policy adjustments. Only through deliberate structural change can clinical trials generate evidence that is both scientifically valid and socially just.

## Data Availability

No new datasets were generated or analyzed during this study. All data supporting the findings of this study are included in the published article and its references.
